# A practical guide to graphic communication for quality assurance, education, and patient care in echocardiography

**DOI:** 10.1111/echo.14464

**Published:** 2019-09-21

**Authors:** Gerald I. Cohen

**Affiliations:** ^1^ Department of Cardiology Ascension St. John Hospital Detroit MI USA

**Keywords:** continuous quality improvement, echocardiography, informatics, quality assurance, telemedicine

## Abstract

Graphic communication (GC) is useful for continuous quality improvement (CQI), education, and patient care when in‐person discussion is not possible because of geographic and schedule constraints. In echocardiography, these constraints can be mitigated by (a) capturing screenshots and device photos or videos and sharing them by email or text message, (b) simultaneous viewing of images on digital displays, and (c) broadcasting the study real time during acquisition to other mobile or stationary devices. Screenshots are useful for CQI and education and can be acquired, annotated, and shared with minimal impact on the flow of clinical echo interpretation. Providers at different locations can employ GC for shared clinical decision making by viewing echo studies from the same server, video conferencing or accessing real‐time broadcast from a device. Which GC tool is selected is determined by its ease of use, the provider's goals and whether immediate image review is needed.

## 1 INTRODUCTION

In‐person communication can be a challenge in a busy echo laboratory because providers may be working in different locations. Furthermore, unscheduled communication may interrupt the flow of patient care and cause inefficiency or errors. However, there are times when shared decision making is needed at a moment's notice but is difficult because of the geographic separation of providers. Options include reviewing images on workstations at different locations or on computers or devices that can access image sharing or broadcasts.^1^, ^2^, ^3^, ^4^, ^5^ If an immediate discussion is not needed, stored or captured videos and still image files or connecting hyperlinks can be shared. This capture and send approach to sharing images allows the recipient to continue with the current clinical work and review the sent information later.^6^, ^7^

This paper describes the different ways of communication with images or graphic communication (GC) can be done and tailored to different settings for continuous quality improvement (CQI), education, and clinical care for echocardiography. However, the same principles may also be useful for other imaging modalities. The focus is first on how to perform CQI by quickly capturing and sharing computer screenshots followed by a review of other established and new GC methods and their applications. In preparing this paper, products of different companies are described for illustrative purposes but without full inclusion of all companies and all possible technologies. Health Insurance Portability and Accountability Act (HIPAA) compliance of a technology depends on appropriate use of the technology, such as adjustment of device or software settings. Subscription to a paid service may be required. The purpose of this review is not to imply an endorsement or warrant assurance of security, privacy, or safety of any product or software.

## 2 SCREENSHOT CAPTURE AND SHARING

Sharing screenshots is a powerful and straightforward way to communicate graphics. The most intuitive way to do this on a computer with a Windows (Microsoft ®) operating system (OS) is to press the keyboard "PrtScn" hotkey. Doing this copies the entire computer display into the OS clipboard. Pressing CTRL‐V into the body of an email program, such as Microsoft® Outlook®, pastes the image into the message. The embedded image generally will need to be resized and possibly cropped. Preparation of the email also includes typed message with comments on the image, a confidentiality message, filling the subject and email address fields, and encryption and privacy settings. Subsequently, the recipient of the email can review the image(s) and message and respond by email or in person at a convenient time. The PrtScn approach is straightforward and does not require additional software. Because the PrtScn key is a hotkey, multiple mouse clicks are not needed to initiate ancillary software to start the process. Disadvantages include the extra time that is needed to resize and crop the captured image, transition between windows (workstation to email program) and paste the image to the email.

Some of the disadvantages of "PrtScn" are overcome by a Microsoft® program called the Snipping Tool that is included with Windows OS Vista and later and listed under "Accessories." Advantages of this software include (a) cropping of the image at the time of selection, which is faster than cropping afterward, (b) limited ability to add annotations directly onto the image, (c) one click opening of Outlook® and pasting the image into the body of an email, and (d) the ability to save the image onto a computer hard drive. The user can more quickly initiate the program by creating a desktop shortcut or a default hotkey (the latter requires modification of the application properties in the associated system folder). Snip & Sketch is a recent update of Snipping Tool with enhanced functionality in Windows 10 that includes a PrtScn hotkey option and enhanced screenshot annotation.

**Figure 1:**
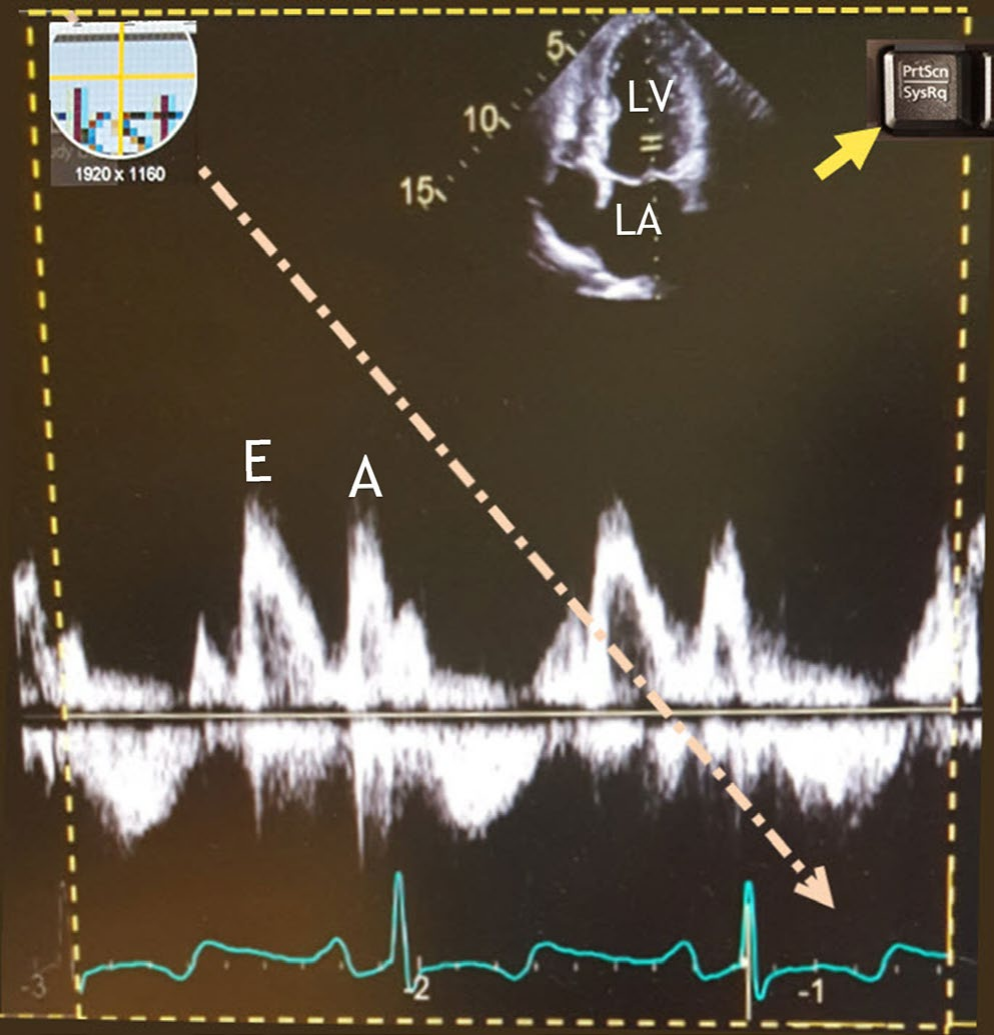
When a hotkey is selected (yellow arrow), a cursor appears that can be dragged over the display (diagonal arrow) to select the region to be captured (dashed outline). In this example, the "PrtScn" hotkey is reassigned to open Snagit®. Early (E) and late (A) diastolic antegrade flow into the left ventricle

**Figure 2:**
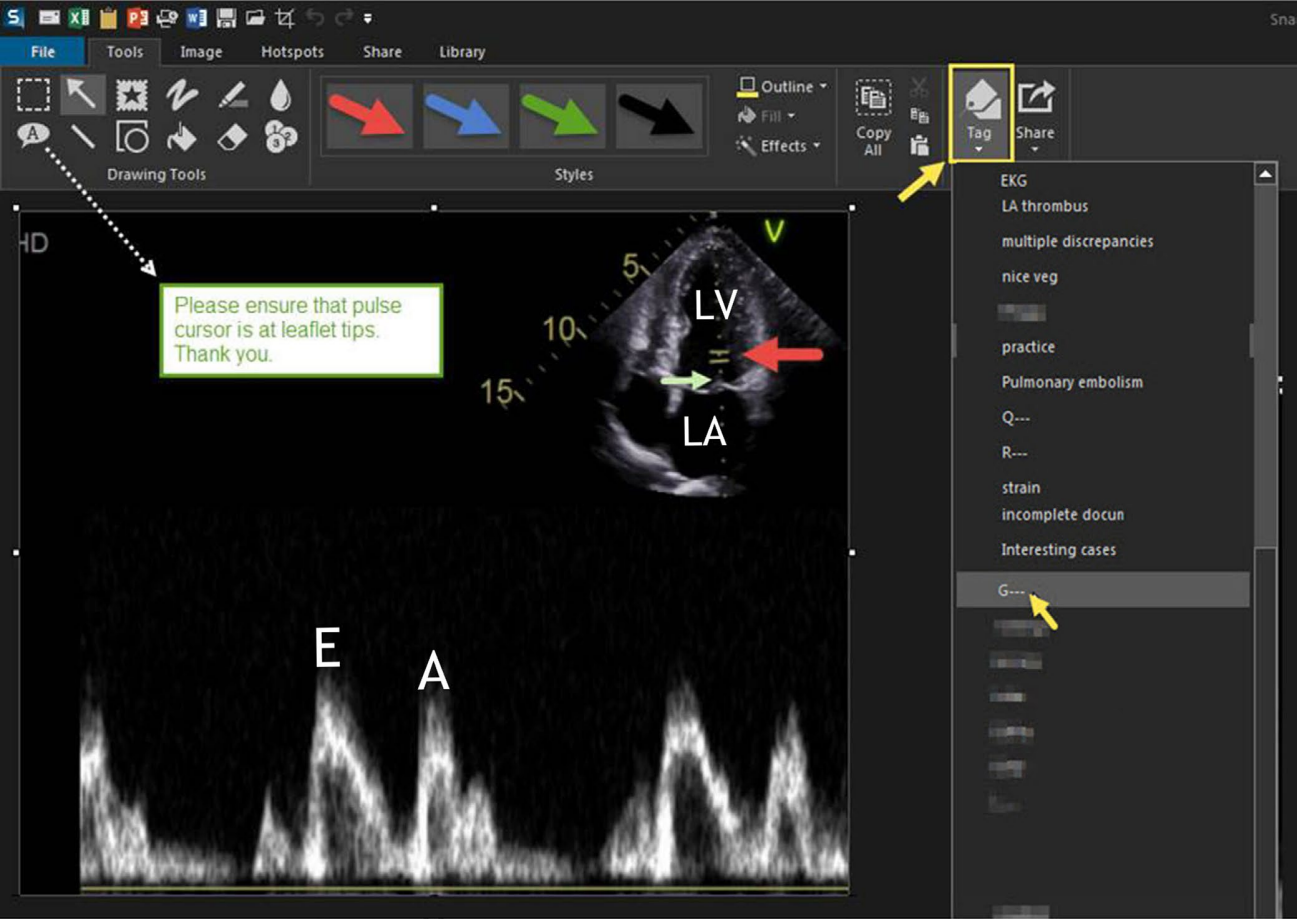
The Snagit® window opens and displays the captured image. Selection of "Tools" from the second from the top toolbar enables annotation. Different annotation types can be selected (third toolbar) and added to the image. These include text, arrows, shapes, and other images. Color coding the arrow red, for error, or green, for correct, can obviate the need for text annotation. For example, a red arrow is added to indicate incorrect positioning of the pulse wave cursor while a green arrow indicates the correct position. Text annotation can also be added (dotted white arrow). One option is to store the image in a library by selecting the tag button (yellow arrow and outline at the right of the third toolbar) to store the image in "G‐‐‐'s" folder (yellow arrow at list folders on the right panel). LA, left atrium; LV, left ventricle, and other abbreviations as in Figure 1

**Figure 3:**
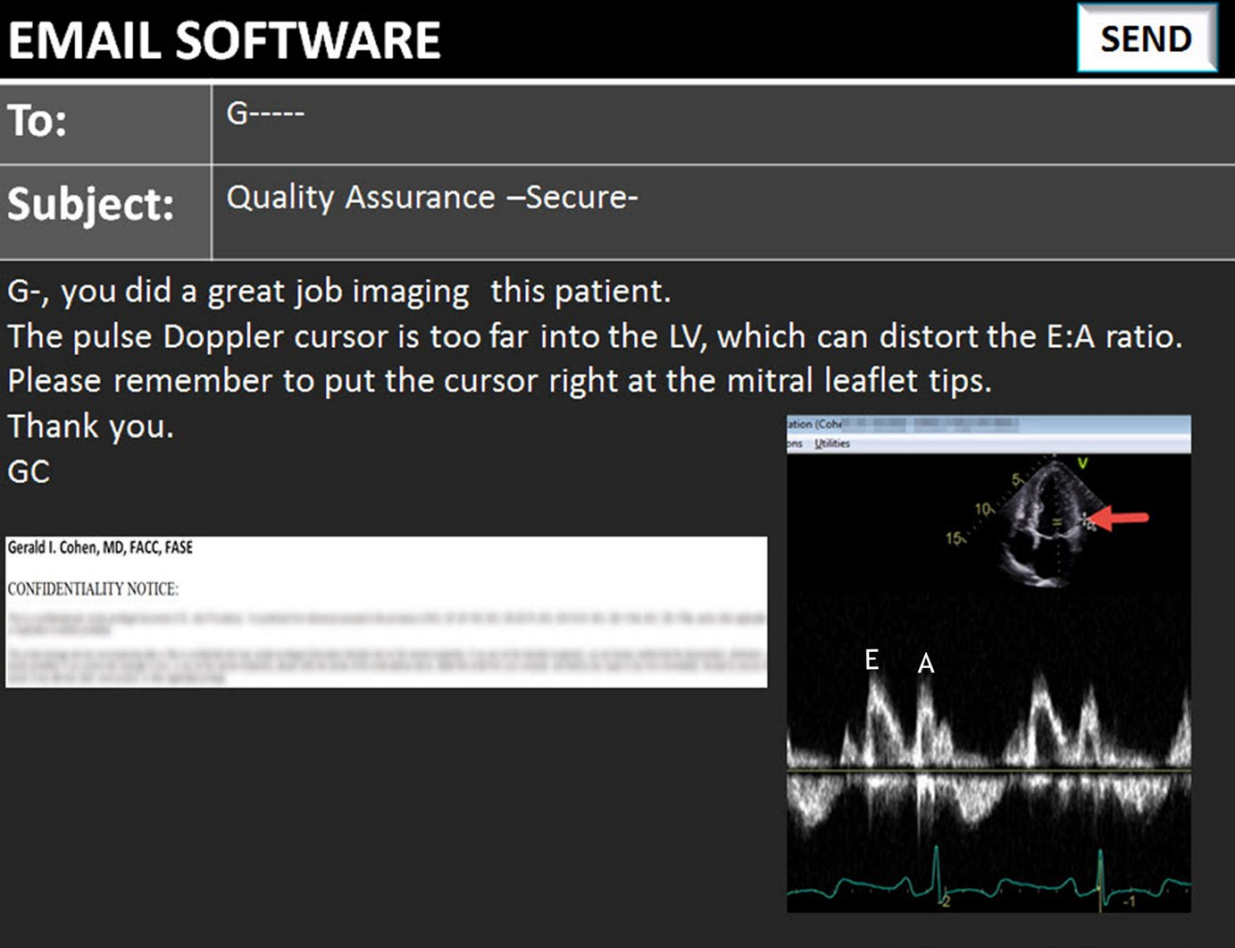
An annotated image is pasted into the body of an email message. The message can include additional comments and images. Videos can also be sent as an attachment (not shown). The email can also be sent to multiple recipients. Confidentiality is achieved by typing a command in the subject field which encrypts the message and by a legal statement at the bottom of the message. Abbreviations as in Figure 1

**Figure 4:**
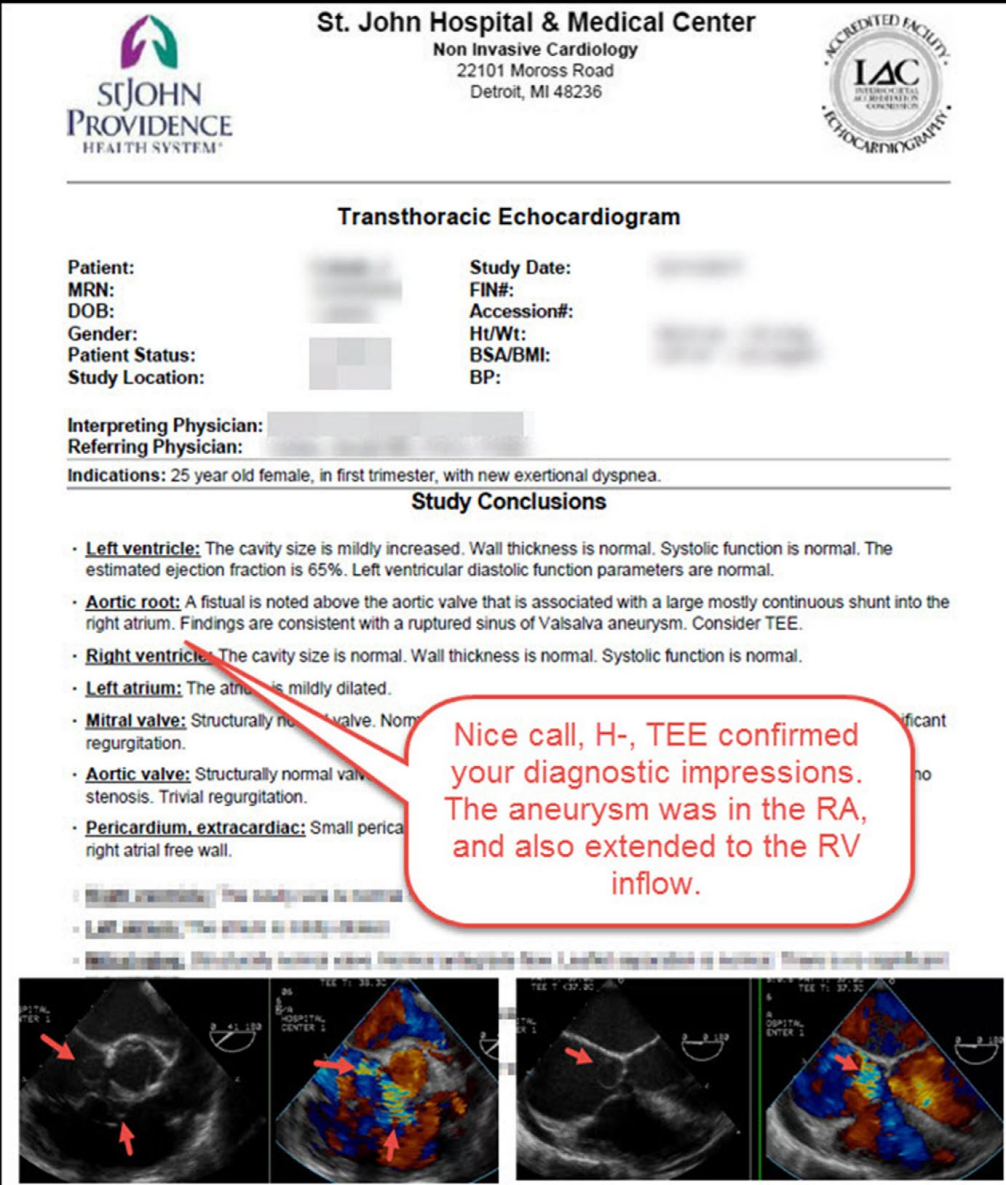
Example of annotation of a transthoracic echocardiographic report in a patient with a sinus of Valsalva aneurysm. The diagnostic impression of the interpreting physician was confirmed during subsequent transesophageal echocardiography. Annotating reports and documents in this way initiates discussion and indicates or explains agreement or disagreement with the recipient of the graphic feedback

An online search of purchasable screenshot programs lists different vendors. For the last five years, we have used a software called Snagit® (TechSmith) to embed cropped images into emails we distribute to sonographers, physicians, and trainees. The process of doing this generally takes 30–60 seconds in conjunction with text macros, such as Autocorrect and signature customization in Outlook® and generates a regular stream of emails without significantly slowing workflow (Movies S1 and S2). Snagit® incorporates a hotkey, selects a cropped image (Figure 1), enables diverse annotations (Figure 2) and, with one mouse click, can open Outlook® and paste the cropped, annotated image into the body of the email message. As with the Window® PrtScn method, standard email fields must be manually filled to ensure the security and privacy of the email before sending it (Figure 3). Captured images can be saved on a computer drive or into the Snagit® library. In our experience, the ability to add diverse annotations like arrows, shapes, and text comments is especially useful for feedback to sonographers regarding measurements and image quality and to physicians regarding report accuracy and completeness (Figure 4).

Snagit® can also crop and capture video on the computer display, but without annotation (which can be done with other tools or software if desired). The captured video can then be saved as an mp4 file and attached to an email message and if desired saved to the computer hard drive or the Snagit® library. Videos take more time to capture and consist of larger files. Advantages include more detailed feedback and education, the illustration of complex or subtle findings, optional inclusion of voice narration, and generation of "how to" guides such as for measurements or operation of the cardiovascular information system (CVIS). Anonymized videos or screenshots can be broadly emailed to disseminate a teaching point to a group of providers.

Health Insurance Portability and Accountability Act (HIPAA) compliance during email exchange and computer access depends on several measures, but most importantly on user adherence to mandatory standards of conduct that assure patient privacy and is a requirement for employment. Other measures include, but are not limited to user registration, password‐protected logon internally, multistep authentication for an external computer or device logon, encryption of emails sent externally, monitoring of all forms of communication, auto‐disconnection during computer inactivity, and no third‐party sharing.

## 3 LIBRARY STORAGE OF IMAGES AND VIDEOS

**Figure 5:**
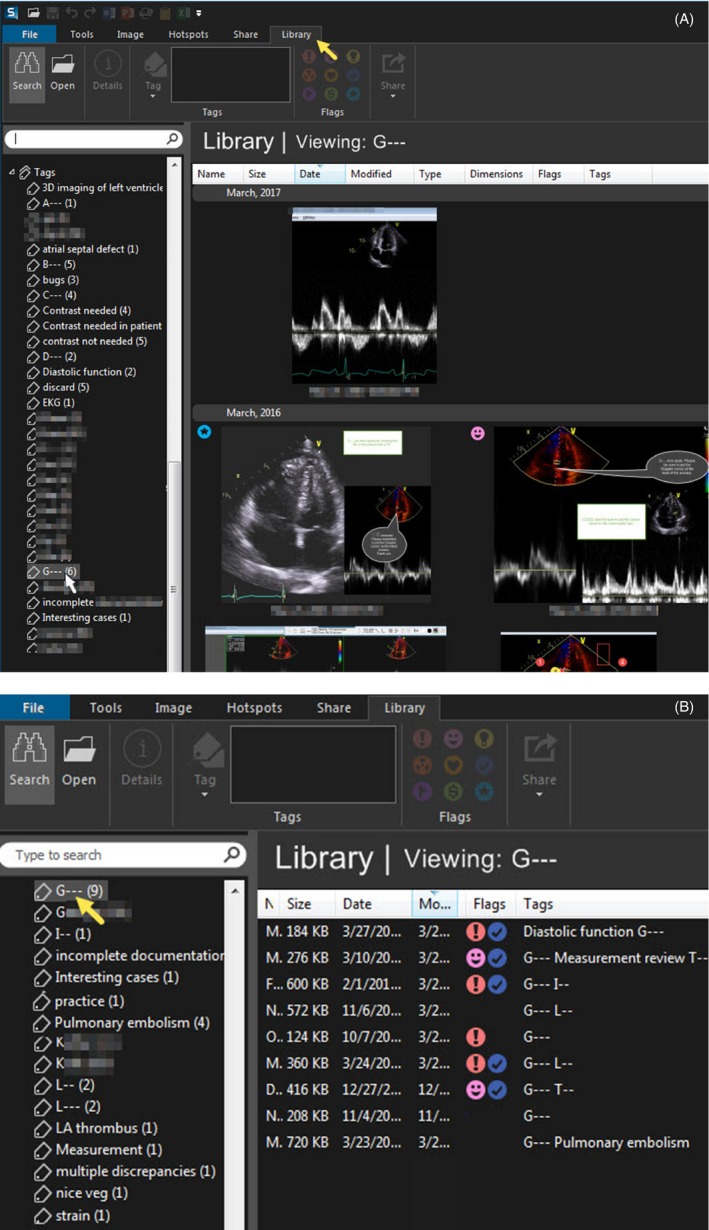
Example of images stored in the Snagit ® "Library" that is accessed by selecting "Library" from the upper toolbar (yellow arrow). Tagged images are stored in folders (left) that can be accessed by a mouse click (arrow on "G‐") which opens the folder to display images and videos in either icon (A) or list (B) views. Review of the contents of a selected folder can facilitate QA review or education. Flags can be added to items in some versions of Snagit®, to guide action planning or provide an overview of achievement

Screenshots can be saved as image files on a hard drive and reviewed later in person or as a group during quality assurance meetings and educational conferences. Software like Snagit® has a built‐in library that streamlines the storage of both still images and videos into customizable folders (Figure 5). Both the Snipping Tool and Snagit® also allow direct saving of the annotated images as jpeg or other image files.

As an alternative to screenshots, some CVIS systems, such as TeraRecon® iNtuition^™^, streamline library storage and database query of flagged images and information. Different criteria can filter this query such as health care provider, patient demographics, clinical data, date range, measurements, and interpretation descriptors. The system also streamlines the generation of emailed feedback by attachment of hyperlinks so that the recipient can securely access images online.

Administrative, physical, and technical safeguards are present to protect the confidentiality and integrity of the protected health information. These safeguards include ensuring the security of the network and using a server or cloud to stores images so that patient‐related data and images are not stored on the local cache or computer. For example, as an FDA‐cleared medical device, TeraRecon® iNtuition^™^ servers and clients are designed and verified in compliance of the guidelines medical centers are required to follow, specifically in relation to controlled, secured, and auditable access. Safeguards include network security (HTTPS as example), role‐based secured access and multi‐layer encryption (SSL as example) as desired and enforced by the customer requirements. Usage logs are available for system administrators and accessible by privilege‐based settings and are exportable for customer audit needs.

## 4 REAL‐TIME GRAPHIC COMMUNICATION

### 4.1 Text messaging

Text messaging via a smartphone or tablet is another way to send graphic feedback. It enables both intermittent and real‐time communication. One way to do this is by downloading HIPAA‐compliant apps such as the Halo Clinical Communication Platform™ (Halo Communications®). With this platform, images and videos can be attached to encrypted messages and sent in real time to other NPI verified colleagues who subscribe to the same service within or outside their health care system. All information stays in the app and cannot be sent or copied to nonverified and nonsubscribing individuals. Real‐time communication has the benefit of securely pairing verbal discussion with images to allow immediate decision making, which can be helpful during routine and acute care for diverse scenarios, such as whether a patient should undergo transesophageal echocardiography. Satisfactory device camera and image resolution are requisite.

### 4.2 Remote workstation review

Providers at different locations may view an echo study on a local CVIS accessible computer and discuss the findings over the phone. This approach is practical but requires each user to separately locate the same study and navigate to the same images. If a specific video frame warrants discussion, each provider must scroll to it independently. The absence of live screen‐sharing prevents mutual and simultaneous viewing of cursor movement, postprocessing, and other manipulations. Verbal communication compensates for this limitation. If the provider uses an undedicated personal computer or wireless device for CVIS login, access and functionality may be limited or slower compared to a dedicated wired workstation at sites that may be at a less accessible location.

**Figure 6:**
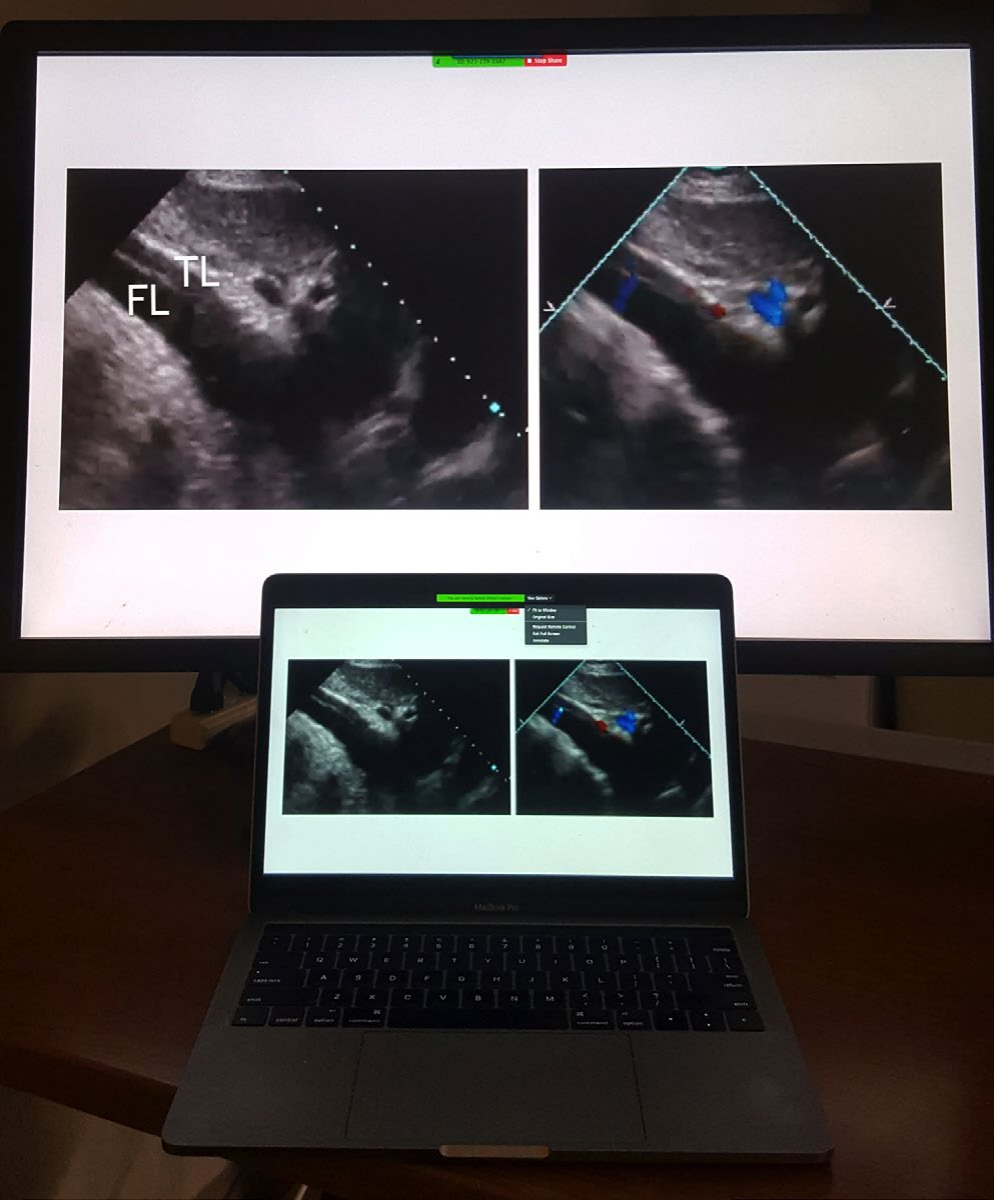
Duplication of images from a desktop workstation (above) via the Internet to a laptop (below) using a video conferencing service that enables HIPAA compliance (Zoom Video Communications, Inc). The subcostal 2D images show aortic dissection with color Doppler flow through an intimal tear in the abdominal aorta. One or more health care providers can view the workstation images on their mobile or remote devices and discuss the findings. Control of the workstation mouse can be passed from one provider to another for image selection, measurement, manipulation or postprocessing. FL, false lumen; TL, True lumen

Video conferencing with a service that enables HIPAA compliance overcomes the above disadvantages by enabling a provider to transmit images on a CVIS workstation to a remote device without CVIS login capability. The service allows transmitting and receiving devices to simultaneously view images and their settings, cursor position, and postprocessing (Figure 6). Also, control of the cursor can be switched from one device to the other, like the passing of a baton which enables the remote device to manipulate the image, change settings, or make measurements akin to robotic control. Advantages include enhanced shared decision making and education. Limitations include possible degradation of image resolution on the remote device and slower video frame rate.

Both the business providing the video conferencing service and the health care provider ensure patient privacy by policies and safeguards that are described at the US Health and Human Services website (http://www.hhs.gov) and established by the signature of a HIPAA business associate agreement (or BAA). Additional safeguards include, but are not limited to assurance that all transmissions have robust (256‐bit AES) end‐to‐end encryption, no persistent storage of protected health information (PHI), disabled cloud recording and the inability of the teleconferencing company to access PHI (Zoom Video Communications, Inc).

### 4.3 Live point‐of‐care ultrasound remote stream

Direct point‐of‐care streaming from the imaging device is a new development that has been achieved by integrating the IIT Reacts collaborative platform (Innovative Imaging Technologies) with the Lumify pocket transducer (PHILIPS). This combination enables live sharing of the ultrasound stream, as well as the bidirectional sharing of audio, webcam video stream, and interactive virtual pointers. The user login connects peer‐to‐peer via a private, encrypted network to protect patient privacy. Ultrasound images that a remote user has access to are de‐identified. Real‐time streaming of ultrasound during acquisition might be useful in some settings, especially if doable with ultrasound machines of all sizes.

A physician on one side of the planet can guide a sonographer or physician in a remote location real time on what images are to be obtained and even how to obtain them and at the same time, discuss the findings, the clinical scenario, and optimal care. A sonographer may want a cardiologist in another location to decide whether image quality warrants the use of echo contrast. Tailoring contrast usage in this way may translate into more appropriate usage in addition to time and cost savings. Other common reasons for video consultation may include cardiac emergencies such as possible myocardial infarction, dissection, and tamponade.

## 5 HISTORICAL PERSPECTIVE AND FUTURE DIRECTIONS

Telemedicine is the embodiment of graphic communication and has evolved dramatically since its inception. Changes include signal digitization, video compression, the advent of wireless options, increased computer speed, miniaturization, power, and increased user knowledge and experience with new technologies. Real‐time transmission enables a remote expert to guide image acquisition, perform interpretation, and discuss findings with providers and patients situated in different geographic locations. The first video satellite transmission occurred during the 1988 Armenian earthquake with the support of the US‐USSR Space‐Bridge project.^1^ In 1989, Finley et al described live, high‐resolution broadcasts of echocardiograms using dedicated telephone cable and analog microwave communication between two distant Canadian maritime hospitals.^2^

Initial telemedicine video transmissions were between two fixed points. Although beneficial to patient care, flexibility is a limitation. The advent of the Internet and wireless communication enabled versatile, low cost, and rapid transmission between multiple access points, which was revolutionary for telemedicine.^1^, ^2^, ^3^, ^5^, ^6^, ^7^ Increased Internet bandwidth enabled the faster transmission of larger stored image files and real‐time transmission of video files of higher image quality and frame rate.^5^ One of the first ways this was done was by pointing a tablet camera at the monitor of the echo cart. In a Brazilian study, doing this enabled real‐time transmission of pediatric images from remote locations to experts in tertiary care centers.^8^ While one may anticipate some image degradation compared to direct echo cart transmission, this process substantially enabled timely congenital heart disease recognition. In the absence of Internet access because of a remote location, commercial satellite transmission remains an option, albeit at an increased cost.^1^, ^4^, ^5^

Device miniaturization has also improved telemedicine access. Choi et al described point‐of‐care imaging at a remote Honduran village by nonexperts, including relief workers. The images were acquired and stored on a pocket‐size ultrasound device and transmitted by dial‐up modem or broadband to a remote workstation for expert interpretation. Diagnostic accuracy was high, even with studies on a smartphone display which were associated with high intraobserver agreement compared to workstation viewing.^9^

Telemedicine goes full circle when it allows the interpreter remote from the patient to control the acquisition of images. One way to do this is to teach basic skills of hands‐on imaging to someone near the patient so that a remotely located provider can verbally guide scanning while viewing the generated images real time. Robotic imaging may be another option though futuristic, expensive, and not necessarily superior to human direction and imaging. Robotic imaging, like robotic surgery, consists of control of a robotic arm that moves the transducer on the patient and is guided by an operator located at a remote location. Different applications have been described, such as carotid imaging.^10^ Other innovations include the ability to remotely operate the controls of the echo cart or imaging device, such as the ability to put in patient data, adjust gain, turn Doppler on or off, and steer the 3D fields of interest. Artificial intelligence may enhance this process by enabling autonomous robotic imaging and potentially on‐site interpretation, such as by estimation of left ventricular ejection fraction.^11^

## 6 CONCLUSION

Graphic communication can be performed in different ways to transcend geographic separation of providers and time and workflow challenges. Taking screenshots is a straightforward, practical approach that is useful for education and CQI, especially when discussion can be deferred. Alternatively, the database of the CVIS system can be recruited to filter, flag, and share images or studies. When immediate communication is needed, providers can review together studies at separate workstations or teleconferenced from a single workstation, send and discuss texted images and videos, and view real‐time acquisition device broadcasts. These tools promise to develop further and enhance the knowledge and the integration of telemedicine with quality assurance and education.

## DISCLOSURES

SofTrek Information Services Inc, Franklin, MI. Lantheus Medical Imaging Inc, Billerica, MA.

## Supporting information


**Movie S1**. How to use screenshots for quality assurance and education.

**Movie S2**. How to simply capture and email a screenshot to send feedback.
